# Mars ain’t the kind of place to raise your kid: ethical implications of pregnancy on missions to colonize other planets

**DOI:** 10.1186/s40504-016-0043-5

**Published:** 2016-08-25

**Authors:** Haley Schuster, Steven L. Peck

**Affiliations:** Biology Department, Brigham Young University, Provo, UT 84602 USA

## Abstract

The colonization of a new planet will inevitably bring about new bioethical issues. One is the possibility of pregnancy during the mission. During the journey to the target planet or moon, and for the first couple of years before a colony has been established and the colony has been accommodated for children, a pregnancy would jeopardize the safety of the crew and the wellbeing of the child. The principal concern with a pregnancy during an interplanetary mission is that it could put the entire crew in danger. Resources such as air, food, and medical supplies will be limited and calculated to keep the crew members alive. We explore the bioethical concerns of near-future space travel.

## Introduction

A non-profit organization called Mars One has begun to sort through candidates to take the nine month journey to Mars and establish a human colony with the goal to make Mars habitable. The colonization of a new planet will inevitably bring about new bioethical issues. One is that the possibility of pregnancy during the mission could jeopardize the safety of the crew and the wellbeing of the child. Mars One has not stated a hard-fast rule about preventing pregnancy, only that it will advise against it. Solutions offered include having the astronauts permanently or temporarily sterilized which would be the most certain way to prevent pregnancy. Mars One’s intention to colonize a planet opens up a number of questions that it is not too early to start exploring. For example, what are the ethical implications of a space program requiring the astronauts to be sterilized? What are the implications of potential reproduction in space? A closer look at human reproduction in light of future space travel, however, is warranted. Especially in light of calls for further consideration of women’s issues in spaceflight (Drudi and Grenon, 2014). We focus especially on the implication of pregnancy during space fight and use Mars One as an illustration for our exploration.

## Pregnancy in space

Pregnancy during a mission such as Mars One is dangerous because it could put the entire crew in danger. Resources such as air, food, and medical supplies will be limited and carefully gauged to keep the crew members alive. An unexpected addition to the crew could put these carefully managed resource and risk calculations out of balance. Moreover, anticipating such a contingency greatly increases the complexity and cost of a mission. A pregnancy and child would put a strain on the mother and compromise her ability to continue her necessary duties for the mission, therefore putting additional workload on the remainder of the crew. Other crew members would be forced to compensate but with few crewmembers (only four crew members are proposed by Mars One) it would still be detrimental to have one of them facing the additional physical challenges of pregnancy especially if there are complications due to the potential for significant risk to the mother’s life. These dangers mean that preventing pregnancy will be critical to the mission, suggesting that individuals selected for the mission may need to be willing to give up their fertility permanently or temporarily for the well-being of the crew and the mission.

Pregnancy in lower gravity environments has been shown to be feasible, but not without challenges. Studies by Ijiri (2004) and Ronca (2003), show that animal pregnancies and births occur in microgravity, indicating that such events could happen in the 40 % gravity on Mars as well. However, mammal births on the International Space Station (ISS) cited by Ronca were found to be more difficult during labor and were more likely to result in stillbirths. This finding implies that a human birth would also be more difficult in microgravity.

Further evidence suggests that environmental factors such as low gravity and the presence of cosmic radiation could have a detrimental effect on the fetus during development. Substantial data has demonstrated that radiation can cause gross malformations, growth retardation, and central nervous system abnormalities in the fetus (Straume, Blattnig, & Zeitlin, 2010).

Space agencies are already well aware of the dangers of cosmic radiation to astronauts and measures are taken to provide adequate shielding, but the effects radiation could have on a fetus have not been well-studied. The total amount of radiation astronauts could potentially experience on their way to Mars was recently estimated by equipment on the Curiosity rover’s spacecraft as it was en route to Mars. The radiation level was estimated to be about 0.66 Sv (Zeitlin et al. 2013). This amount falls within the range in which the risk of birth defects increases substantially (McCollough et al. 2007). Additionally, solar flare events can occur and temporarily increase the radiation dose even if there is adequate shielding for normal conditions. If a Martian base were established, the radiation on the surface of Mars could be even more problematic since space suits cannot shield as much radiation as a habitat structure or spacecraft could, so an astronaut who is pregnant would need to restrict the time she spends outside. Although, a pregnant female astronaut would not likely fit EVA suits provided should emergency use be needed.

In addition to the health concerns of a pregnant astronaut there is evidence that decreased gravity negatively affects fetal development. Experiments performed with pregnant rats in the NASA Space Shuttle provide evidence that lack of gravity disrupts the development of the vestibular system (Ronca et al. 2008). This finding means a child conceived during the flight to Mars could have problems balancing and orienting him or herself on the surface of Mars. Studies using human bone marrow stem cells found a significant effect on cells in microgravity and fewer cells were generated (Davis et al., 1996). Since cell proliferation was decreased in these human stem cells, it is likely that the space environment could cause the fetus to develop abnormally (e.g., see also Zhang et al. 2016; Beck et al., 2012; Ogneva, 2015). A child with birth defects, or one born prematurely, would be more difficult to care for in an environment with such limited resources. This suggests that bearing a healthy baby is less likely on such a mission, and therefore the negative consequences of pregnancy suggest this possibility should be actively mitigated against.

## The health of mother and crew

Pregnancy on the mission would also pose a greater risk to the mother. Although one or two of the astronauts will receive comprehensive medical training and medical equipment to treat anticipated illnesses or injuries will be available, the crew will likely not be prepared to assist with birth complications or provide care for a newborn. Should something go wrong during the pregnancy or birth, the crew may not have the skills or supplies necessary to keep the mother and child safe. Further, the confined environment of the ship and the lack of gravity could pose additional challenges for neonatal care. The wellbeing of the mother and child could be greatly compromised if complications should arise. Surgeries have been completed successfully on rats in microgravity (Campbell Mark et al. 2005), however the time in surgery was longer compared with surgeries in Earth gravity. Moreover, despite the success of these surgeries, it seems likely that microgravity would pose other unexpected challenges for human surgery. For example, it has also been shown that microorganisms thrive in microgravity environments (Horneck et al. 2010). Factors such as disease virulence, infectability, and transference in microgravity are largely understudied. In addition, resistance factors of disease organisms might also be modified in space. Researchers suggest that pregnant women may be at greater risk for contracting an illness in space (Santy & Jennings, 1992).

Pathogens may spread among the crew more easily in the cramped conditions of a spacecraft. The observation that astronauts’ immune systems are suppressed due to microgravity is well established (Martinez et al. 2015) and it has been shown that “immune system dysregulation occurs during flight” (Sams et al. 2015). NASA researchers expect that an impaired immune system could pose a major risk for longer space flights, such as the one to Mars. Connections between stress levels and changes in the immune system’s ability to react properly are well documented, which suggests that the extra stress a pregnancy would surely cause will likely have a negative effect on the mother’s immune system. With an increased risk to the mother, it would be better to take measures to prevent pregnancy for the health of the mother, as well as the greater good of the crew and the mission.

## Sex in space

Human sex drive has been found to persist when humans are isolated in a small group for an extended time. At this time, NASA’s policy forbids sex in space, and there have been no confirmed instances of it happening, but the lengthy trip to Mars and even starting a colony could result in sex occurring. There have been seven recorded pregnancies and more undisclosed due to privacy policies in remote Antarctic research stations, where small crews are isolated together for long months in similarly stressful and dangerous environments (Stuster, 2011). This is unexpectedly high for a group of highly trained, professional scientists. Thus, with a mixed gender crew, sexual intercourse is likely during the years of the crew’s isolation on a mission to colonize Mars or any such long term space exploration. Perhaps an effective training program could inspire crew members to place solidarity of the group ahead of personal interests. Such a perspective, if possible to maintain, would greatly reduce the occurrences of sexual activity, but it cannot guarantee abstinence. Therefore, extra precautions for such a long-duration mission are necessary.

Pregnancy must have been enough of a problem or concern that in 2011, the U.S. Antarctic Program instituted a new rule requiring all women of childbearing years to take a pregnancy test before being cleared to live in Antarctica. Their reasoning for prohibiting pregnant women is

“Because clinics at U.S. stations are not equipped or staffed to provide adequate prenatal care, manage obstetric emergencies, or perform abortions, medical evacuation may be necessary. There are few transportation options during the isolated Antarctic winter. Consequently, pregnancy puts not only the mother and unborn child at risk but also the flight crews and other station personnel.” (United States Antarctic Program).

With long-duration space travel, such as the trip to Mars, these same concerns would be even more important because evacuating or receiving necessary pregnancy-related supplies would not be an option. Since there is no way to evacuate a pregnant woman during a trip to Mars, preventing a pregnancy from even occurring becomes crucial, thus the importance of temporary or permanent sterilization. Upon the announcement of this new policy, some feared that this would result in women being treated differently or unfairly in the hiring process (Carmon, 2011). Similar concerns would arise from a requirement for astronauts to be temporarily or permanently sterilized.

Terrestrial space flight analogs, that is, isolated confined environments such as Navy ships and submarines can provide insight into this kind of problem. In the Navy, it has been reported that pregnancies occurring while on duty on ships make up less than one percent of the Total Force (Daniel, 2013). While this means there is only a small number of pregnancies that occur in such situations, it does show that pregnancies do in fact occur in such stressful, confined, rigid environments. According to the Navy press release cited above, the low percentage is due to the educational programs the Navy provides about contraception methods and family planning. It is probably also safe to assume that getting pregnant while on operational duty is highly discouraged in their training. But, despite all the training the Navy provides, pregnancies do still occur. One study performed by the Center for Naval Analysis reported that every year a significant number of unplanned personnel losses from Navy ships are due to pregnancy (Garcia, 1999), although the actual number is not available. This data is for the whole Navy, on ships with much bigger crews than the four person Mars crew, but it does provide information that pregnancies do happen in high stress, confined environments such as those on Navy ships.

The issue of a pregnancy on a submarine has only come to light recently because only a few countries allowed women to serve on submarines (Kane and Horn 2001). Starting in 2016, women will be serving on United States Navy submarines. This new development has been met with controversy and opposition. Some of the arguments against adding women to submarine crews involve the possibility of women getting pregnant during tours of duty. One concern is the potential contamination of the filtered air and the presence of high levels of carbon dioxide, both of which could be harmful to a developing fetus (Kane and Horn 2001). At present, the effects of submarine air on fetus development have not been studied (Kane and Horn 2001). Mars spaceships and colonies will similarly be totally confined, self-sustaining environments, so contaminated air and high levels of carbon dioxide could be a problem for them as well.

While these comparable situations can be helpful in predicting potential issues in a Mars trip, the consequences of a pregnancy are not as great. Pregnant women in these situations can be removed from isolated and dangerous situations, and can go home. A rescue mission for astronauts would take longer than a full term pregnancy to orchestrate. Further, the dangers associated with a pregnancy are not as high in an Earth gravity environment. Therefore, the consequences of using less effective contraceptive measures are not as great. Astronauts on a Mars mission will need to use more effective methods.

## Contraception in space

There is a possibility that it could be easier to become pregnant in microgravity. Radiation exposure will likely reduce sperm count, but there is evidence that suggests sperm cells swim faster in microgravity. One study found that sperm is affected by small changes in gravity and found that fertilization in hypergravity was slowed down (Tash, Kim, Schuber, Seibt, & Kinsey, 2001). A similar study in microgravity, or even reduced gravity, still needs to be performed, but Tash, et al. propose that an opposite effect may occur in microgravity. If fertilization could potentially occur faster, other contraceptive methods may not be able to work as well, and therefore sterilization may be a more effective choice.

Table 1 shows the success rate of various forms of contraception (Contraception, 2015). The most effective methods of contraception are sterilization and long-acting reversible contraception, which essentially amounts to temporary sterilization. Sterilization methods, including permanent and temporary, are the only birth control methods besides abstinence that are nearly one hundred percent effective (Suszynski 2014) each with less than 1 % failure rate (Contraception, 2015).

**Table 1:** Failure rate of various forms of contraception (Contraception, 2015)

Birth control method	Failure rate
Implant	0.05 %
Male sterilization (vasectomy)	0.15 %
Levonorgestrel IUD	0.2 %
Female sterilization (tubal ligation)	0.5 %
Copper Intrauterine Device (IUD)	0.8 %
Injection (“shot”)	6 %
Oral contraceptives (“the pill”)	9 %
Patch	9 %
Hormonal vaginal contraceptive ring	9 %
Diaphragm	12 %
Male condom	18 %
Female condom	21 %
Spermicides	28 %

Permanent methods include vasectomy and female tubal ligations, and even hysterectomy. Reversals of vasectomies and tubal ligations are possible, but they are costly and there is a low success rate of regaining fertility.

Temporary sterilization methods include the copper intrauterine device (IUD), progesterone IUD, and progesterone implant. These can last for ten years, three or five years, and 3 years, respectively. Depending on how long it takes a proposed colony to be ready for children, the temporary methods may or may not last long enough, and replacements may be needed. All other birth control methods rely on human responsibility to regulate their effectiveness. The next best option after the sterilization methods is the progestin shot given every three months and has a typical use failure rate of 6 %. This and other methods such as condoms or “the pill” are less effective (ranging from 9 to 28 % failure rate) and require supplies and check-ups that may not be available on the mission, whereas sterilization is a onetime procedure. The supplies and packaging that would be required for non-sterilization contraception would take up precious cargo space and may not even retain its effectiveness for the length of time needed; especially for the Mars One plan. Further, it is not known if the constant bombardment of radiation on the contraceptives would decrease their effectiveness or not.

Despite the overwhelming evidence supporting the necessity of sterilization on a Mars mission, specific ethical arguments have been advanced against the idea of sterilization and even contraceptives in general, and similar arguments could be formed against this requirement. In most ethical traditions, the right to reproduce is a fundamental freedom. Requiring permanent or temporary sterilization would infringe upon the right of each astronaut to reproduce. The right to choose which medical procedures are performed on one’s body is also commonly upheld, and requiring astronauts to go through the process of sterilization or use the temporary methods may hinder that right as well. This policy may feel discriminatory against those that want to retain their fertility or have personal objections to undergoing the procedures or using the temporary methods. To avoid forcing astronauts into doing something that goes against their personal beliefs, it will be important for these requirements to be clearly laid out from the time they are put in place. That way, a person who does not wish to fulfill the requirement of becoming infertile may choose a different path. The necessity of being infertile during a long duration space flight is so essential for the safety of the whole crew, that the astronauts will have to give up their right to reproduce freely. While this requirement may seem drastic or harsh, it will be necessary to prevent the crew members from endangering each other.

Common arguments against contraceptives include claims that it goes against the goals of a Mars colony, is anti-life, prevents people who could benefit humanity from being born, leads to immoral behavior, and that it goes against many religious beliefs. In this case, Mars astronauts may only need to be sterilized for a decade or so before a colony is set up well for children, although the colony’s child-readiness could likely happen beyond their lifetimes or fertility years. Regarding arguments that contraceptives are effective enough, the catastrophic consequences of a mission pregnancy suggest that sterilization is necessary to preserve the lives of the crew by preventing an unexpected increase in resource requirements. The children born would have a higher risk of intellectual and physical disabilities which would strain resources. Further, it’s unlikely that sexual activity can be prevented. It is also possible that extended periods of radiation on the surface of Mars may eventually make the astronauts sterile; it is a risk inherent to the mission.

Those who see sterilization as morally wrong either from religious or deontological perspectives may have to carefully consider participation in such a mission. Those with such views do not have to be a part of the mission and they should not prevent others from taking this safety precaution. The catastrophic nature of a pregnancy on such a mission suggests that all crew members must take the necessary precautions to protect against this possibility. The question still remains, however, if the responsibility of avoiding pregnancy lies on all crew members, or only the ones capable of becoming pregnant. If the goal is to decrease the chance of any crew member becoming pregnant as much as possible, then it would follow that all crew members must be permanently or temporarily infertile. The group as a whole needs to be infertile. Sterilization would not be necessary for single sex crews or crews with women who have undergone menopause.

Based on the evidence gathered thus far, sterilization appears to be the best choice of action for preventing pregnancy during preliminary missions to Mars. A utilitarian approach seems mandated to reduce negative consequences for the group as a whole. Therefore, ethically, it seems desirable to require astronauts to be sterilized. Temporary or permanent, sterilization is a more certain way to prevent the negative effects a pregnancy could have on the entire crew such as danger to the mother, abnormalities in the fetus, and limited resources for the crew, and to better ensure the safety and well-being of all.

## Conclusions

We have not discussed a number of other considerations that will require future work. For example, the psychological stresses and harms that might come from a pregnancy in space have not been considered. Others may want to further explore the ethical question of requiring permanent or temporary sterilization from the perspective of different ethical perspectives such as differing religious orientations. One related ethical question that should be explored is whether it is ethical to require the burden of pregnancy prevention to fall only on women. Additionally, how can the mission planners avoid burdening only women with the responsibility of contraception if there are no equally effective temporary contraceptive methods for men. Furthermore, the medical effects of sterilization on women are more substantial and invasive, while vasectomy is known to be safe and effective. Finally, if a woman were to conceive mid-mission, how would current debates about abortion play out and should medical procedures and resources be in place to make this option available? More research needs to be done to better understand the dangers of pregnancy on such a long duration space mission. Space agencies considering a mission to Mars (to visit or colonize) will want to consider these and other ethical questions in order to better ensure the safety and well-being of their crews.
